# Supplementary material for: Isolation of Cysteine-Rich Peptides from Citrullus colocynthis
Source: Biomolecules. 2020 Sep 16;10(9):1326. doi: 10.3390/biom10091326 (PMC7565491; doi:10.3390/biom10091326)
Supplement: Supplementary file 1 [file biomolecules-10-01326-s001.zip › biomolecules-917147-supplementary-proofreading/Supplementary Materials.pdf]

Isolation of cysteine-rich peptides from *Citrullus colocynthis*

Behzad Shahin-Kaleybar, Ali Niazi, Alireza Afsharifar, Ghorbanali Nematzadeh, Reza Yousefi, Bernhard Retzl, Roland Hellinger, Edin Muratspahić, Christian W. Gruber

Supplementary Materials

Table S1. Comparison of citcol-8 with CRPs from *Momordica spp.*

Gene	Sequence[1]	Uniprot ID	Cys loops pattern	Species
citcol-8	--NRVGVCLFVGKPCWSDADCPSGCYCKPLPLIDAGYCGFL	-	$CX_6CX_5CX_3CX_1CX_{10}C$[2]	*Citrullus colocynthis*
MCoTI-I	SGSDGGVCPKILQRCRRDSDCPGACICRG-----NGYCG--	P82408	$CX_6CX_5CX_3CX_1CX_5C$	*Momordica cochinchinensis*
MCoTI-II	SGSDGGVCPKILKKCRRDSDCPGACICRG-----NGYCG--	P82409	$CX_6CX_5CX_3CX_1CX_5C$	*Momordica cochinchinensis*
EETI-II	------GCPRILMRCKQDSDCLAGCVCGP-----NGFCGSP	P12071	$CX_6CX_5CX_3CX_1CX_5C$	*Momordica elaterium*
MCoTI-III	----QRACPRILKKCRRDSDCPGECICKE-----NGYCG--	P82410	$CX_6CX_5CX_3CX_1CX_5C$	*Momordica cochinchinensis*

[1]cysteine residues are highlighted in yellow; [2]X represents any amino acid residue except cysteine.

Figure S1. The m/z value list of the b- and y-ions series for the precursor masses used for *de novo* sequencing of citcol-2. The m/z value for b/y ions produced in tandem MS for each of the precursor masses have been shown in seperate Tables. A, B, C and D correspond to detected b/y ions for 3279.2, 822.4, 1723.8 and 1858.6 m/z precursor masses respectively. All the ion masses have been represented as monoisotopic mass signals $[M+H]^{+}$.

A

#	b	Seq.	y	#(+1)
1	-	P	3279.2	28
2	258.0	C	3182.2	27
3	-	W	3022.2	26
4	531.1	S	2836.1	25
5	646.2	D	2749.0	24
6	-	A	2634.0	23
7	832.2	D	2563.0	22
8	992.2	C	2447.9	21
9	-	P	2287.9	20
10	-	S	2190.9	19
11	1233.7	G	2103.9	18
12	1393.3	C	2046.8	17
13	1556.4	Y	1886.8	16
14	1716.4	C	1723.8	15
15	1844.5	K	1563.8	14
16	1941.6	P	1435.7	13
17	2054.7	L	-	12
18	2151.7	P	1225.5	11
19	2264.8	L	-	10
20	2377.9	I	1015.4	9
21	2492.9	D	902.3	8
22	2563.9	A	787.3	7
23	2621.0	G	716.2	6
24	2784.0	Y	-	5
25	2944.0	C	-	4
26	3001.1	G	-	3
27	3148.1	F	-	2
28	3279.2	L	-	1

B

#	b	Seq.	y	#(+1)
1	-	V	822.4	7
2	260.0	C	723.3	6
3	373.1	L	563.3	5
4	520.2	F	450.2	4
5	619.3	V	303.2	3
6	676.3	G	204.1	2
7	804.4	K	-	1

C

#	b	Seq.	y	#(+1)
1	-	C	1723.8	15
2	289.1	K	1563.8	14
3	386.1	P	1435.7	13
4	499.2	L	1338.6	12
5	596.3	P	1225.5	11
6	709.3	L	1128.5	10
7	822.4	I	1015.4	9
8	937.4	D	902.3	8
9	1008.5	A	787.3	7
10	1065.5	G	716.2	6
11	1228.6	Y	659.2	5
12	1388.6	C	496.2	4
13	1445.6	G	-	3
14	1592.7	F	-	2
15	1705.8	L	-	1

D

#	b	Seq.	y	#(+1)
1		V	1858.6	16
2		G	1759.5	15
3	258.1	K	1702.5	14
4	382.2	P	1574.4	13
5	542.2	C	1477.4	12
6	728.3	W	1317.4	11
7	815.3	S	1131.3	10
8	930.3	D	1044.2	9
9	1001.4	A	929.2	8
10	1116.4	D	858.2	7
11	1276.4	C	743.2	6
12	1373.5	P	583.1	5
13	1460.5	S		4
14	1517.5	G		3
15	1677.5	C		2
16	1840.6	Y		1

Figure S2. ***De novo*** **sequencing of citcol peptides (citcol-1 to 7) by MALDI-TOF/TOF.** Proteolytic digestion sites and tandem MS fragmentation spectra including observed b- and y-ion series are shown for citcol-1 (A), citcol-2 (B), citcol-3 (C), citcol-4 (D), citcol-5 (E), citcol-6 (F), citcol-7 (G) and citcol-8 (H). The digestion site by each enzyme has been shown by arrow and the produced fragments used as precursor masses have been shown in bracket in monoisotopic masses $[M+H]^+$. The amino acid sequence was determined by manually fragments assembly of digested fragments and N-terminal b-ions / C-terminal y-ions series identification. Tryptic digestion was used to distinguish between Isobaric amino acids Gln/Lys and chymotrypsin for Leu/Ile. The m/z value list of each of the precursor masses used for used for *de-novo* sequencing by tandem MS have been shown in tables neXt to spectra. Only digestion sites have been shown for the fragments that were sufficient for sequencing the entire peptide.

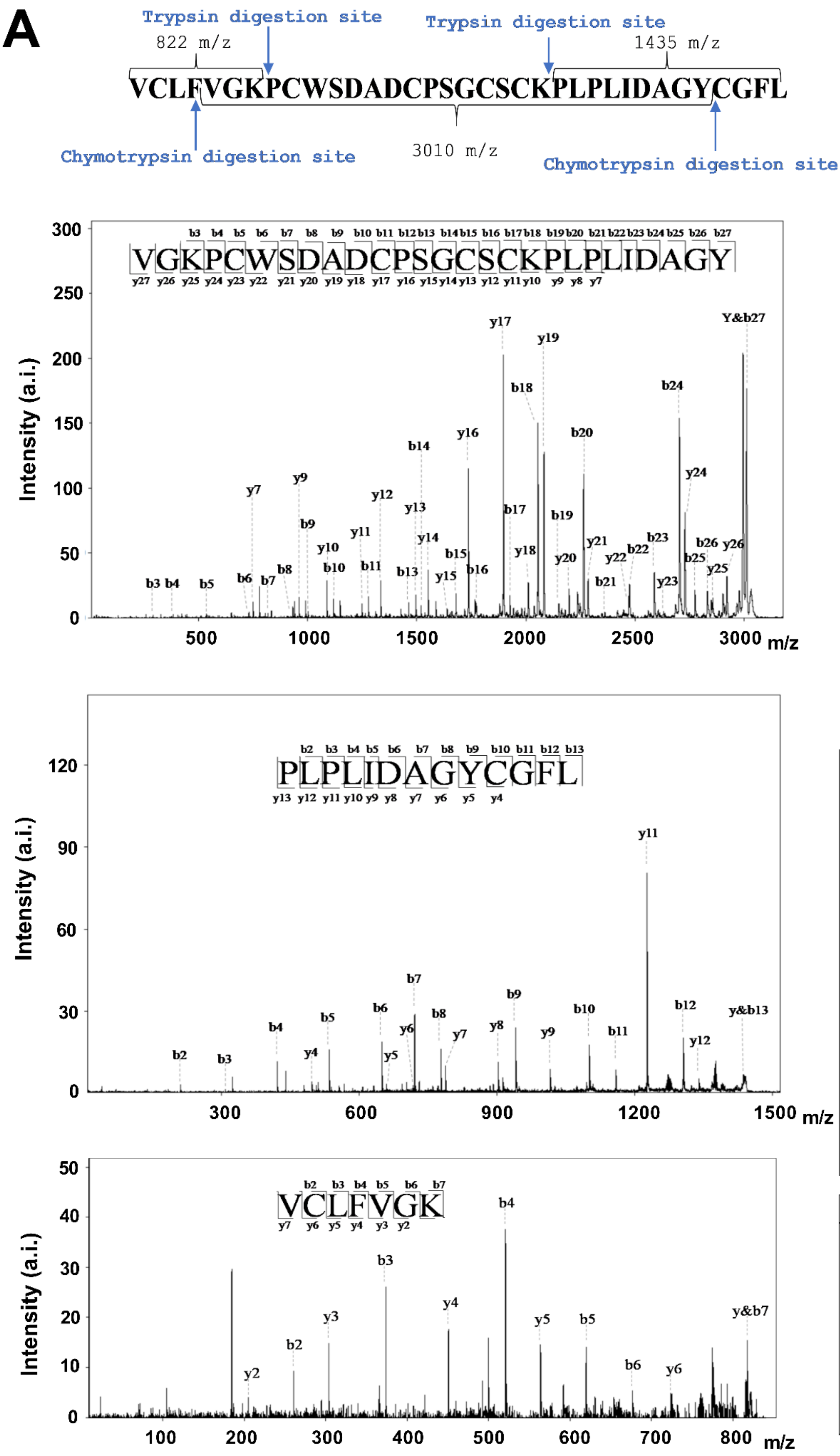

#	b	Seq.	y	#(+1)
1	-	V	3010.2	27
2	-	G	2911.1	26
3	285.1	K	2854.1	25
4	382.2	P	2726.0	24
5	542.2	C	2628.9	23
6	728.3	W	2468.9	22
7	815.3	S	2282.9	21
8	930.3	D	2195.8	20
9	1001.4	A	2080.8	19
10	1116.4	D	2009.8	18
11	1276.4	C	1894.7	17
12	1373.5	P	1734.7	16
13	1460.5	S	1637.7	15
14	1517.5	G	1550.6	14
15	1677.5	C	1493.6	13
16	1764.6	S	1333.6	12
17	1924.6	C	1246.6	11
18	2052.7	K	1086.6	10
19	2149.7	P	958.5	9
20	2262.8	L	861.4	8
21	2359.9	P	748.3	7
22	2472.9	L	-	6
23	2586.0	I	-	5
24	2701.1	D	-	4
25	2772.1	A	-	3
26	2829.1	G	-	2
27	3010.2	Y	-	1

#	b	Seq.	y	#(+1)
1	-	P	1435.7	13
2	211.1	L	1338.6	12
3	308.1	P	1225.5	11
4	421.2	L	1128.5	10
5	534.3	I	1015.4	9
6	649.3	D	902.3	8
7	720.4	A	787.3	7
8	777.4	G	716.2	6
9	940.5	Y	659.2	5
10	1100.5	C	496.2	4
11	1157.5	G	-	3
12	1304.6	F	-	2
13	1417.6	L	-	1

#	b	Seq.	y	#(+1)
1	-	V	822.4	7
2	260.0	C	723.3	6
3	373.1	L	563.3	5
4	520.2	F	450.2	4
5	619.3	V	303.2	3
6	676.3	G	204.1	2
7	804.4	K	-	1

B

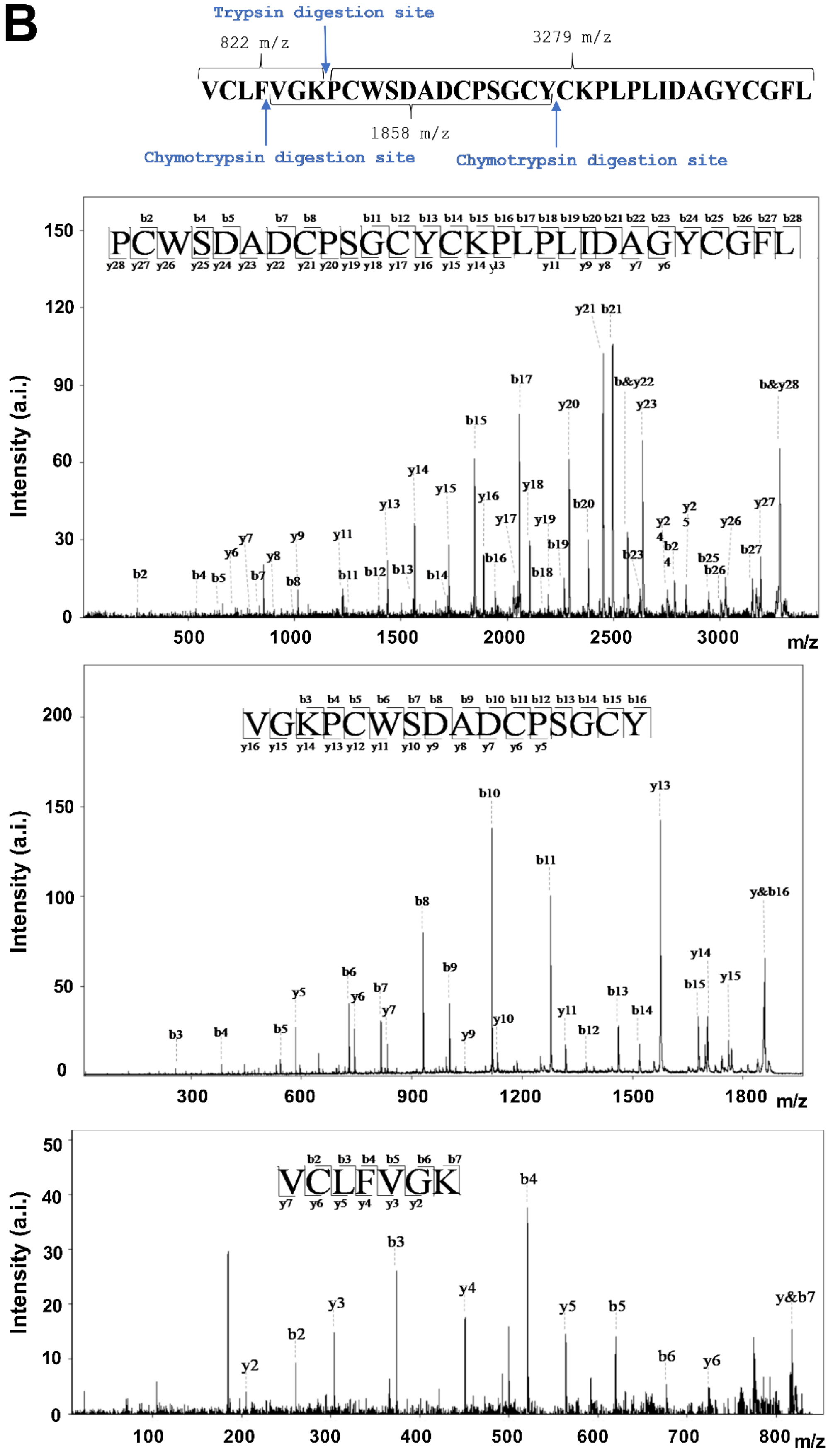

#	b	Seq.	y	#(+1)
1	-	P	3279.2	28
2	258.0	C	3182.2	27
3	-	W	3022.2	26
4	531.1	S	2836.1	25
5	646.2	D	2749.0	24
6	-	A	2634.0	23
7	832.2	D	2563.0	22
8	992.2	C	2447.9	21
9	-	P	2287.9	20
10	-	S	2190.9	19
11	1233.7	G	2103.9	18
12	1393.3	C	2046.8	17
13	1556.4	Y	1886.8	16
14	1716.4	C	1723.8	15
15	1844.5	K	1563.8	14
16	1941.6	P	1435.7	13
17	2054.7	L	-	12
18	2151.7	P	1225.5	11
19	2264.8	L	-	10
20	2377.9	I	1015.4	9
21	2492.9	D	902.3	8
22	2563.9	A	787.3	7
23	2621.0	G	716.2	6
24	2784.0	Y	-	5
25	2944.0	C	-	4
26	3001.1	G	-	3
27	3148.1	F	-	2
28	3279.2	L	-	1

#	b	Seq.	y	#(+1)
1	-	V	1858.6	16
2	-	G	1759.5	15
3	258.1	K	1702.5	14
4	382.2	P	1574.4	13
5	542.2	C	1477.4	12
6	728.3	W	1317.4	11
7	815.3	S	1131.3	10
8	930.3	D	1044.2	9
9	1001.4	A	929.2	8
10	1116.4	D	858.2	7
11	1276.4	C	743.2	6
12	1373.5	P	583.1	5
13	1460.5	S	-	4
14	1517.5	G	-	3
15	1677.5	C	-	2
16	1840.6	Y	-	1

#	b	Seq.	y	#(+1)
1	-	V	822.4	7
2	260.0	C	723.3	6
3	373.1	L	563.3	5
4	520.2	F	450.2	4
5	619.3	V	303.2	3
6	676.3	G	204.1	2
7	804.4	K	-	1

C

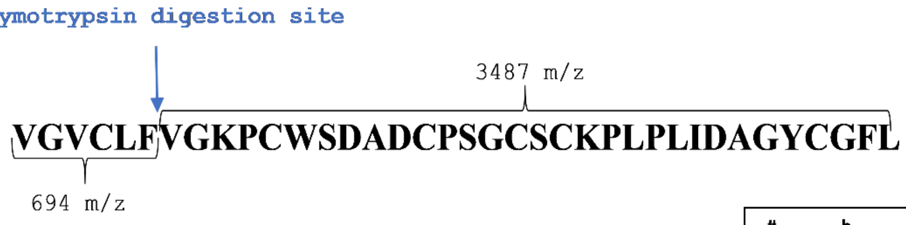

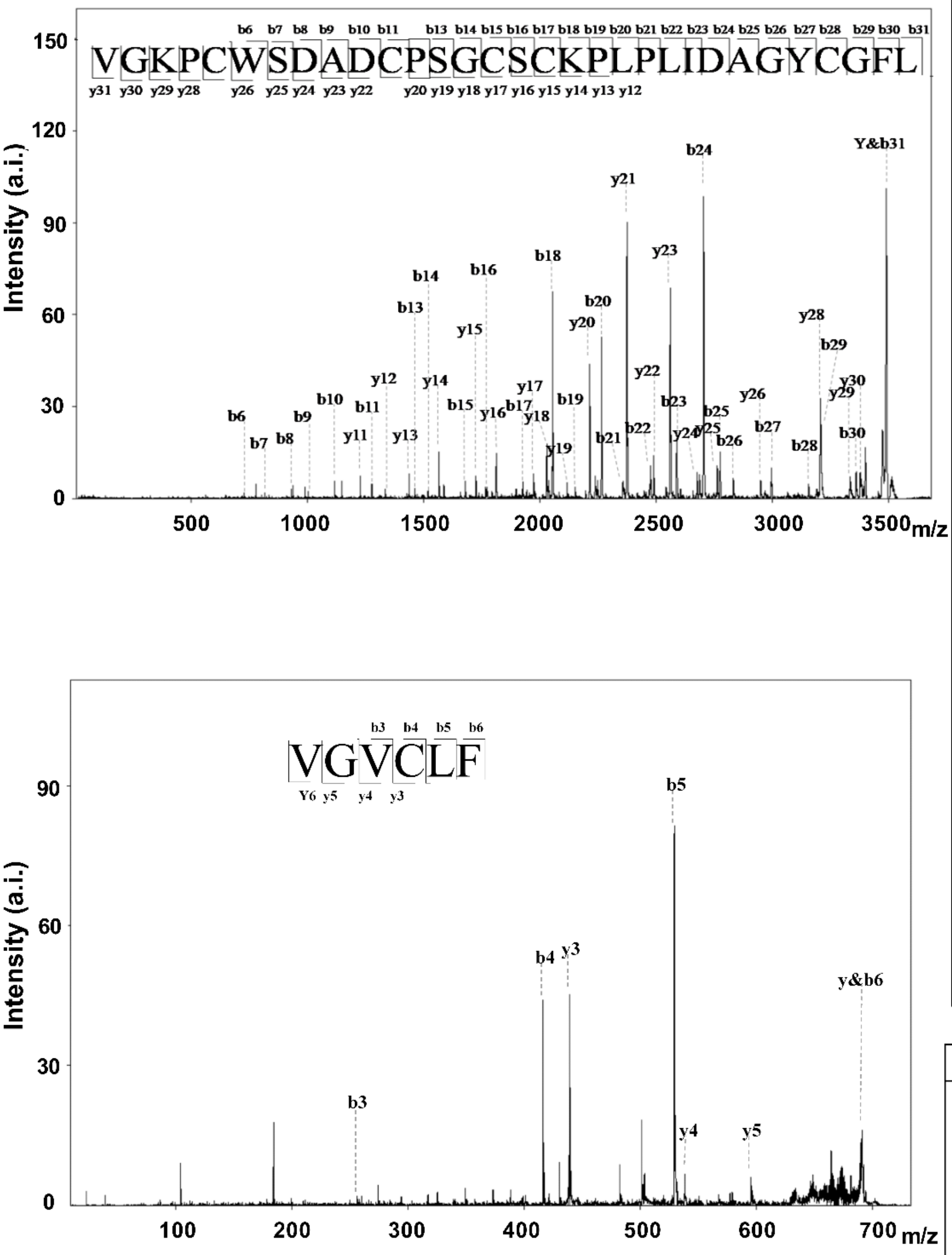

#	b	Seq.	y	#(+1)
1	-	V	3487.4	31
2	-	G	3388.3	30
3	-	K	3331.3	29
4	-	P	3203.2	28
5	-	C	-	27
6	728.3	W	2946.1	26
7	815.3	S	2760.0	25
8	930.3	D	2673.0	24
9	1001.4	A	2558.0	23
10	1116.4	D	2486.9	22
11	1276.4	C	-	21
12	-	P	2211.9	20
13	1460.5	S	2114.9	19
14	1517.5	G	2027.8	18
15	1677.5	C	1970.8	17
16	1764.6	S	1810.8	16
17	1924.6	C	1723.8	15
18	2052.7	K	1563.8	14
19	2149.7	P	1435.7	13
20	2262.8	L	1338.6	12
21	2359.9	P	1225.5	11
22	2472.9	L	-	10
23	2586.0	I	-	9
24	2701.1	D	-	8
25	2772.1	A	-	7
26	2829.1	G	-	6
27	2992.2	Y	-	5
28	3152.2	C	-	4
29	3209.2	G	-	3
30	3356.3	F	-	2
31	3469.4	L	-	1

#	b	Seq.	y	#(+1)
1	-	V	694.3	6
2	-	G	595.2	5
3	256.1	V	538.2	4
4	416.1	C	739.1	3
5	529.2	L	-	2
6	676.3	F	-	1

D

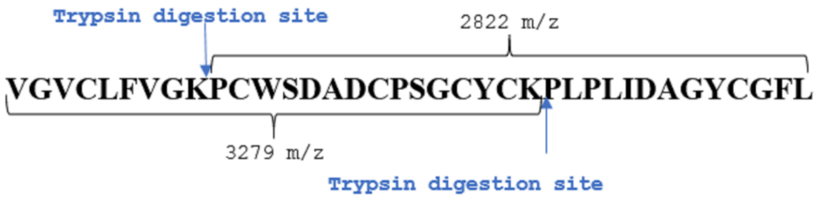

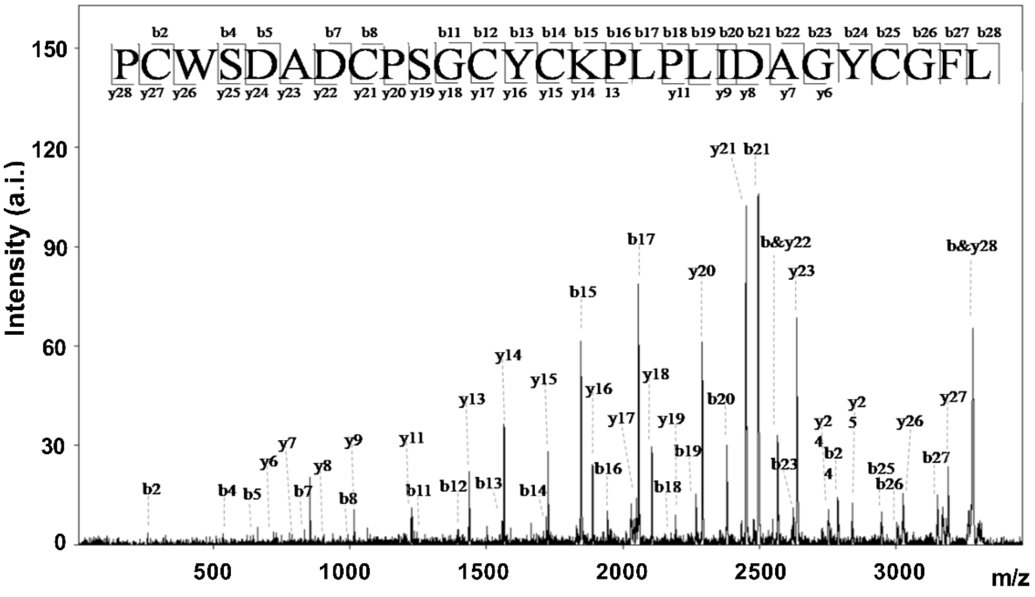

#	b	Seq.	y	#(+1)
1	-	P	3279.2	28
2	258.0	C	3182.2	27
3	-	W	3022.2	26
4	531.1	S	2836.1	25
5	646.2	D	2749.0	24
6	-	A	2634.0	23
7	832.2	D	2563.0	22
8	992.2	C	2447.9	21
9	-	P	2287.9	20
10	-	S	2190.9	19
11	1233.7	G	2103.9	18
12	1393.3	C	2046.8	17
13	1556.4	Y	1886.8	16
14	1716.4	C	1723.8	15
15	1844.5	K	1563.8	14
16	1941.6	P	1435.7	13
17	2054.7	L	-	12
18	2151.7	P	1225.5	11
19	2264.8	L	-	10
20	2377.9	I	1015.4	9
21	2492.9	D	902.3	8
22	2563.9	A	787.3	7
23	2621.0	G	716.2	6
24	2784.0	Y	-	5
25	2944.0	C	-	4
26	3001.1	G	-	3
27	3148.1	F	-	2
28	3279.2	L	-	1

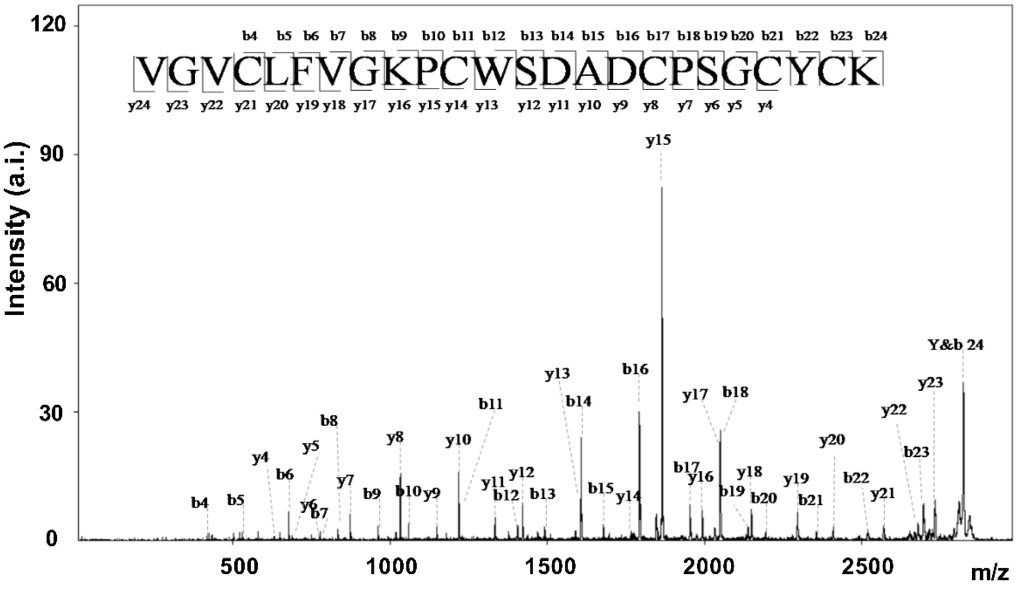

#	b	Seq.	y	#(+1)
1	-	V	2822.0	24
2	-	G	2723.0	23
3	-	V	2665.9	22
4	416.1	C	2566.9	21
5	529.2	L	2406.9	20
6	676.3	F	2293.8	19
7	775.3	V	2146.7	18
8	832.4	G	2047.6	17
9	960.5	K	1990.6	16
10	1057.5	P	1862.5	15
11	1217.5	C	1765.5	14
12	1403.6	W	1605.5	13
13	1490.6	S	1419.4	12
14	1605.7	D	1332.4	11
15	1676.7	A	1217.3	10
16	1791.7	D	1146.3	9
17	1951.7	C	1031.3	8
18	2048.8	P	871.3	7
19	2135.8	S	774.2	6
20	2192.8	G	687.2	5
21	2352.9	C	630.1	4
22	2515.9	Y	-	3
23	2675.9	C	-	2
24	2804.0	K	-	1

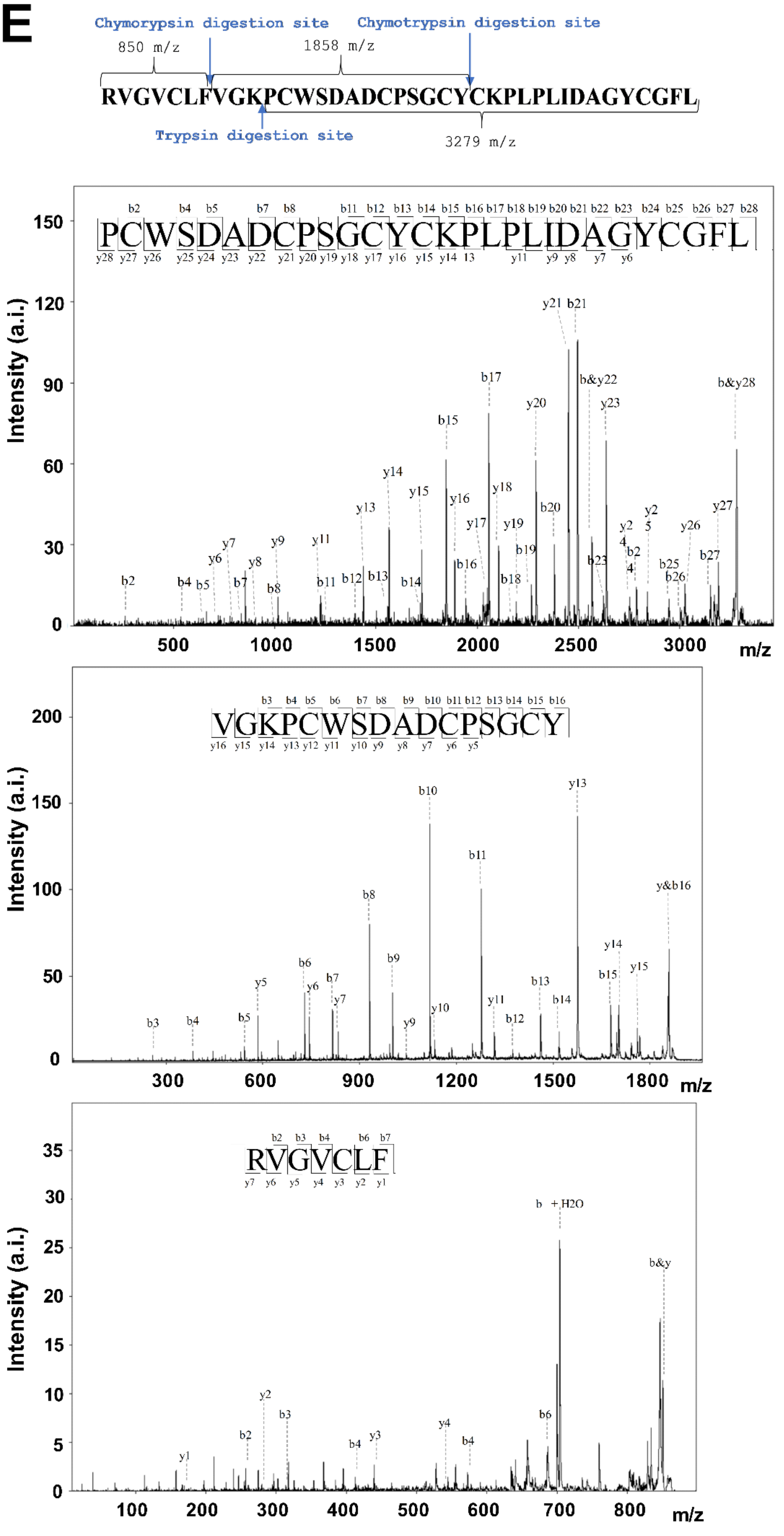

#	b	Seq.	y	#(+1)
1	-	P	3279.2	28
2	258.0	C	3182.2	27
3	-	W	3022.2	26
4	531.1	S	2836.1	25
5	646.2	D	2749.0	24
6	-	A	2634.0	23
7	832.2	D	2563.0	22
8	992.2	C	2447.9	21
9	-	P	2287.9	20
10	-	S	2190.9	19
11	1233.7	G	2103.9	18
12	1393.3	C	2046.8	17
13	1556.4	Y	1886.8	16
14	1716.4	C	1723.8	15
15	1844.5	K	1563.8	14
16	1941.6	P	1435.7	13
17	2054.7	L	-	12
18	2151.7	P	1225.5	11
19	2264.8	L	-	10
20	2377.9	I	1015.4	9
21	2492.9	D	902.3	8
22	2563.9	A	787.3	7
23	2621.0	G	716.2	6
24	2784.0	Y	-	5
25	2944.0	C	-	4
26	3001.1	G	-	3
27	3148.1	F	-	2
28	3279.2	L	-	1

#	b	Seq.	y	#(+1)
1	-	V	1858.654	16
2	-	G	1759.586	15
3	258.192	K	1702.565	14
4	382.24	P	1574.470	13
5	542.254	C	1477.417	12
6	728.333	W	1317.408	11
7	815.365	S	1131.328	10
8	930.392	D	1044.296	9
9	1001.429	A	929.269	8
10	1116.456	D	858.2327	7
11	1276.465	C	743.205	6
12	1373.518	P	583.196	5
13	1460.550	S	-	4
14	1517.571	G	-	3
15	1677.581	C	-	2
16	1840.644	Y	-	1

#	b	Seq.	y	#(+1)
1	-	R	850.4	7
2	256.1	V	694.3	6
3	313.1	G	595.2	5
4	412.2	V	538.2	4
5	-	C	439.1	3
6	685.3	L	279.1	2
7	850.4	F	166.0	1

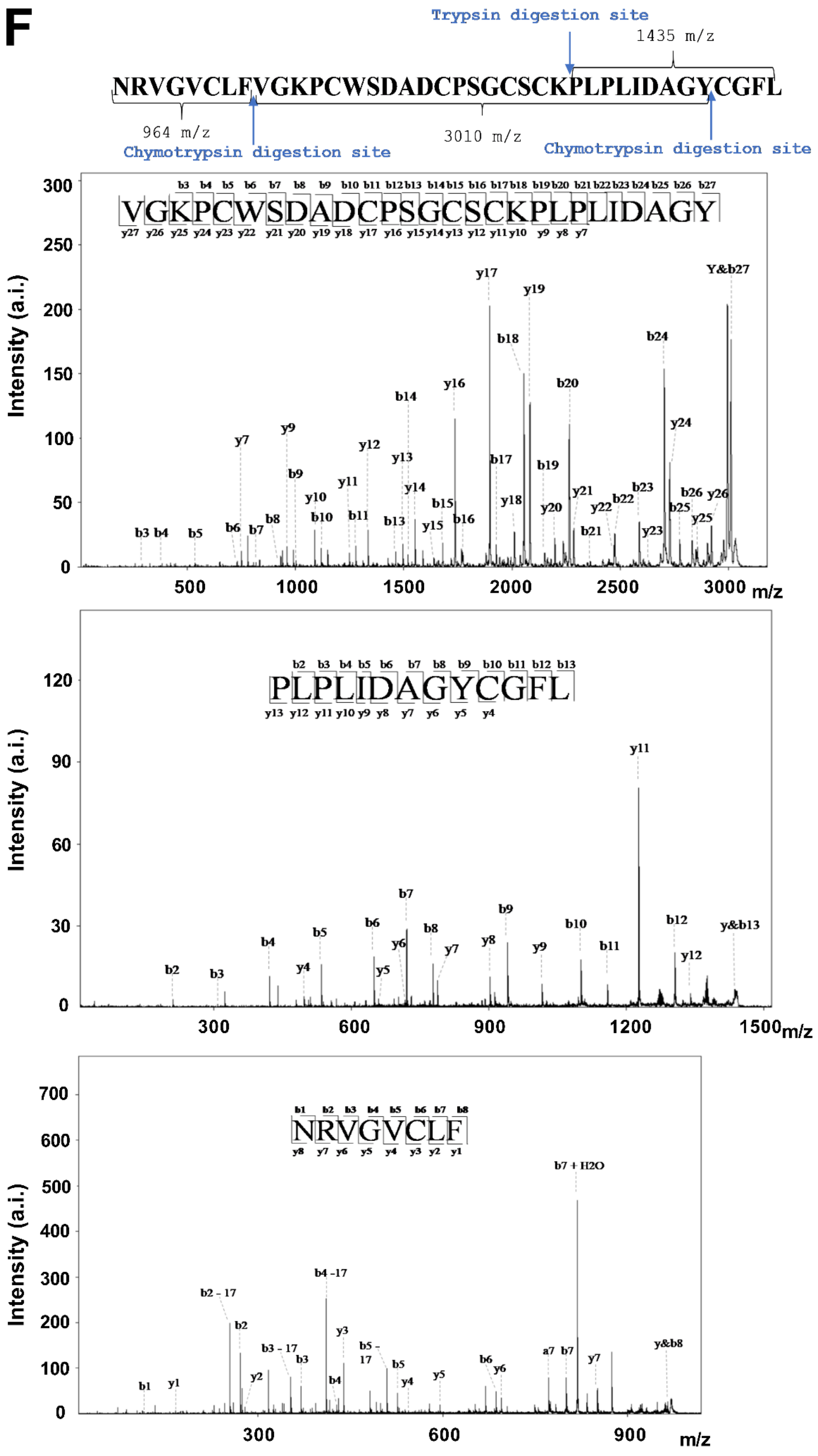

#	b	Seq.	y	#(+1)
1	-	V	3010.2	27
2	-	G	2911.1	26
3	285.1	K	2854.1	25
4	382.2	P	2726.0	24
5	542.2	C	2628.9	23
6	728.3	W	2468.9	22
7	815.3	S	2282.9	21
8	930.3	D	2195.8	20
9	1001.4	A	2080.8	19
10	1116.4	D	2009.8	18
11	1276.4	C	1894.7	17
12	1373.5	P	1734.7	16
13	1460.5	S	1637.7	15
14	1517.5	G	1550.6	14
15	1677.5	C	1493.6	13
16	1764.6	S	1333.6	12
17	1924.6	C	1246.6	11
18	2052.7	K	1086.6	10
19	2149.7	P	958.5	9
20	2262.8	L	861.4	8
21	2359.9	P	748.3	7
22	2472.9	L	-	6
23	2586.0	I	-	5
24	2701.1	D	-	4
25	2772.1	A	-	3
26	2829.1	G	-	2
27	3010.2	Y	-	1

#	b	Seq.	y	#(+1)
1	-	P	1435.7	13
2	211.1	L	1338.6	12
3	308.1	P	1225.5	11
4	421.2	L	1128.5	10
5	534.3	I	1015.4	9
6	649.3	D	902.3	8
7	720.4	A	787.3	7
8	777.4	G	716.2	6
9	940.5	Y	659.2	5
10	1100.5	C	496.2	4
11	1157.5	G	-	3
12	1304.6	F	-	2
13	1417.6	L	-	1

#	b	Seq.	y	#(+1)
1	115.050	N	964.481	8
2	271.151	R	850.438	7
3	370.219	V	694.337	6
4	427.241	G	595.269	5
5	526.309	V	538.247	4
6	686.318	C	439.179	3
7	799.402	L	279.170	2
8	946.471	F	166.086	1

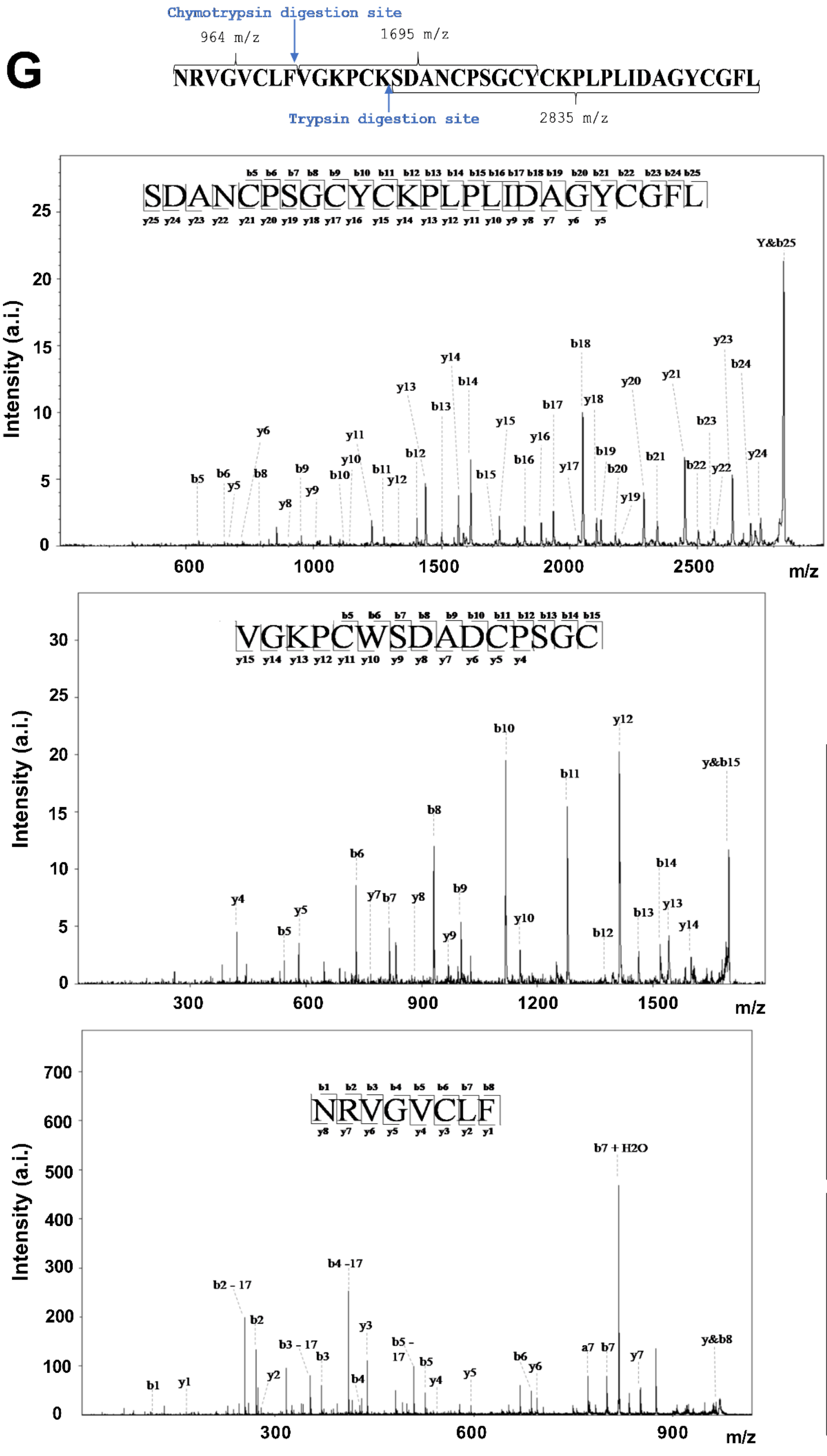

#	b	Seq.	y	#(+1)
1	-	S	2835.1	25
2	-	D	2748.1	24
3	-	A	2633.0	23
4	-	D	2562.0	22
5	549.1	C	2447.0	21
6	646.1	P	2287.0	20
7	733.2	S	2189.9	19
8	790.2	G	2102.9	18
9	950.2	C	2045.9	17
10	1113.3	Y	1885.8	16
11	1273.3	C	1722.8	15
12	1401.4	K	1562.8	14
13	1498.4	P	1434.7	13
14	1611.5	L	1337.6	12
15	1708.6	P	1224.5	11
16	1821.6	L	1127.5	10
17	1934.7	I	1014.4	9
18	2048.8	N	901.3	8
19	2119.8	A	787.3	7
20	2176.8	G	716.2	6
21	2339.9	Y	659.2	5
22	2499.9	C	-	4
23	2556.5	G	-	3
24	2704.0	F	-	2
25	2817.1	L	-	1

#	b	Seq.	y	#(+1)
1	-	V	1695.5	15
2	-	G	1596.5	14
3	-	K	1539.5	13
4	-	P	1411.4	12
5	542.2	C	1314.3	11
6	728.3	K	1154.3	10
7	815.3	S	968.2	9
8	930.3	D	881.2	8
9	1001.4	A	766.2	7
10	1116.4	N	695.1	6
11	1276.4	C	580.1	5
12	1373.5	P	420.1	4
13	1460.5	S	-	3
14	1517.5	G	-	2
15	1677.5	C	-	1

#	b	Seq.	y	#(+1)
1	115.050	N	964.481	8
2	271.151	R	850.438	7
3	370.219	V	694.337	6
4	427.241	G	595.269	5
5	526.309	V	538.247	4
6	686.318	C	439.179	3
7	799.402	L	279.170	2
8	946.471	F	166.086	1

H

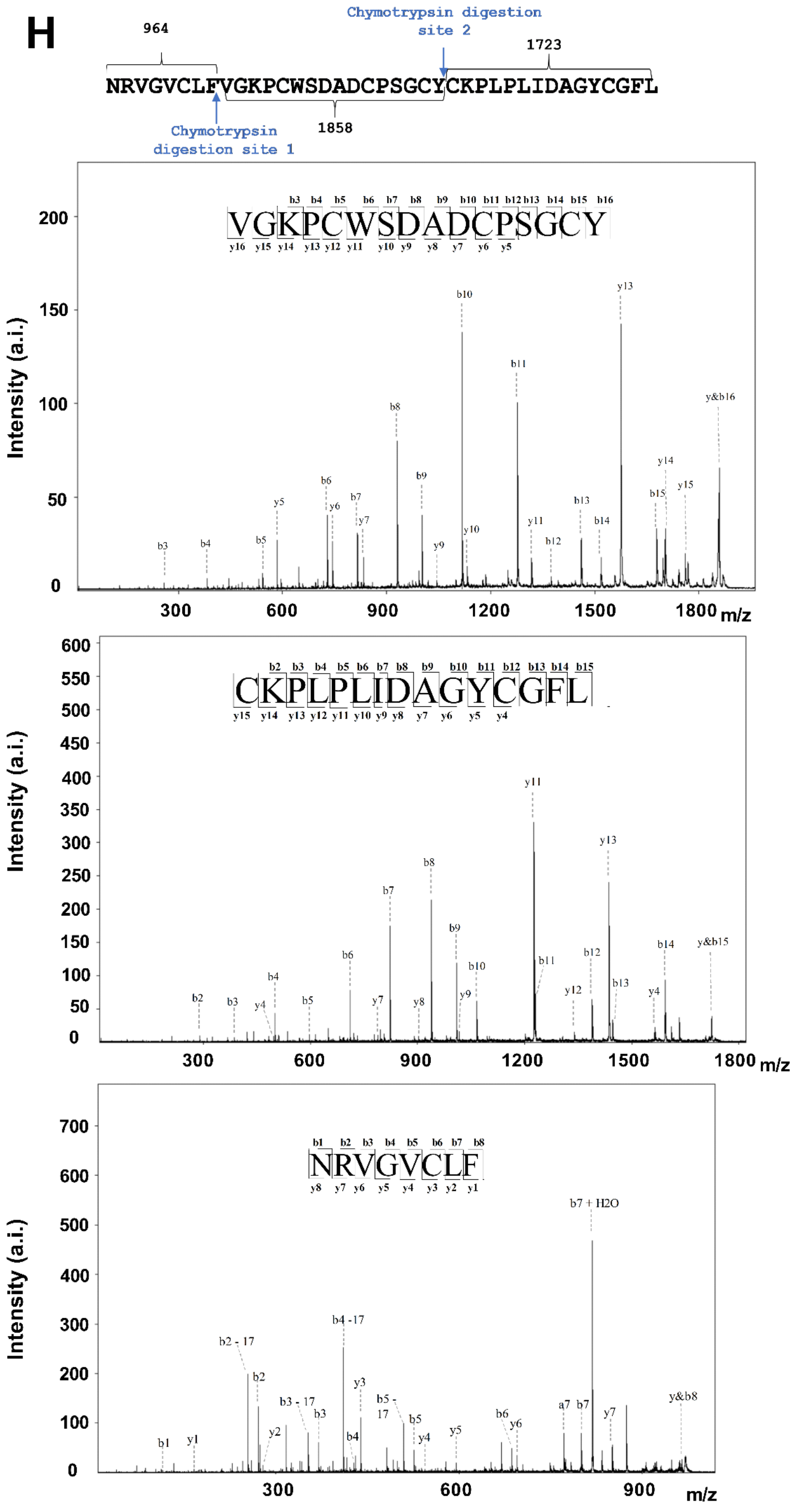

#	b	Seq.	y	#(+1)
1	-	V	1858.654	16
2	-	G	1759.586	15
3	258.192	K	1702.565	14
4	382.24	P	1574.470	13
5	542.254	C	1477.417	12
6	728.333	W	1317.408	11
7	815.365	S	1131.328	10
8	930.392	D	1044.296	9
9	1001.429	A	929.269	8
10	1116.456	D	858.2327	7
11	1276.465	C	743.205	6
12	1373.518	P	583.196	5
13	1460.550	S	-	4
14	1517.571	G	-	3
15	1677.581	C	-	2
16	1840.644	Y	-	1

#	b	Seq.	y	#(+1)
1	-	C	1723.8	15
2	289.1	K	1563.8	14
3	386.1	P	1435.7	13
4	499.2	L	1338.6	12
5	596.3	P	1225.5	11
6	709.3	L	1128.5	10
7	822.4	I	1015.4	9
8	937.4	D	902.3	8
9	1008.5	A	787.3	7
10	1065.5	G	716.2	6
11	1228.6	Y	659.2	5
12	1388.6	C	496.2	4
13	1445.6	G	-	3
14	1592.7	F	-	2
15	1705.8	L	-	1

#	b	Seq.	y	#(+1)
1	115.050	N	964.481	8
2	271.151	R	850.438	7
3	370.219	V	694.337	6
4	427.241	G	595.269	5
5	526.309	V	538.247	4
6	686.318	C	439.179	3
7	799.402	L	279.170	2
8	946.471	F	166.086	1

Figure S3. Enzyme inhibition assays of citcol peptides (200 µM each). (A) Inhibitory activity of selected citcol peptides (1, 2, 7 and 8) against α-amylase enzyme of porcine (*Sus scrofa*), fungi (*Aspergillus oryzae*) and bacteria (*Bacillus subtilis*). (B, C) Inhibitory activity of citcol peptides against α-chymotrypsin (B) and trypsin (C) enzymes. Negative controls (-ve) without peptide or inhibitor (100% enzyme activity) and positive controls (+ve) with known inhibitors (complete inhibition of activity) acarbose for α-amylase, PMSF for α-chymotrypsin and sun-flower trypsin inhibitor (SFTI) for trypsin, respectively, were tested. All the assays were carried out in three replicates and are illustrated as percentage of inhibition (mean±STDEV).

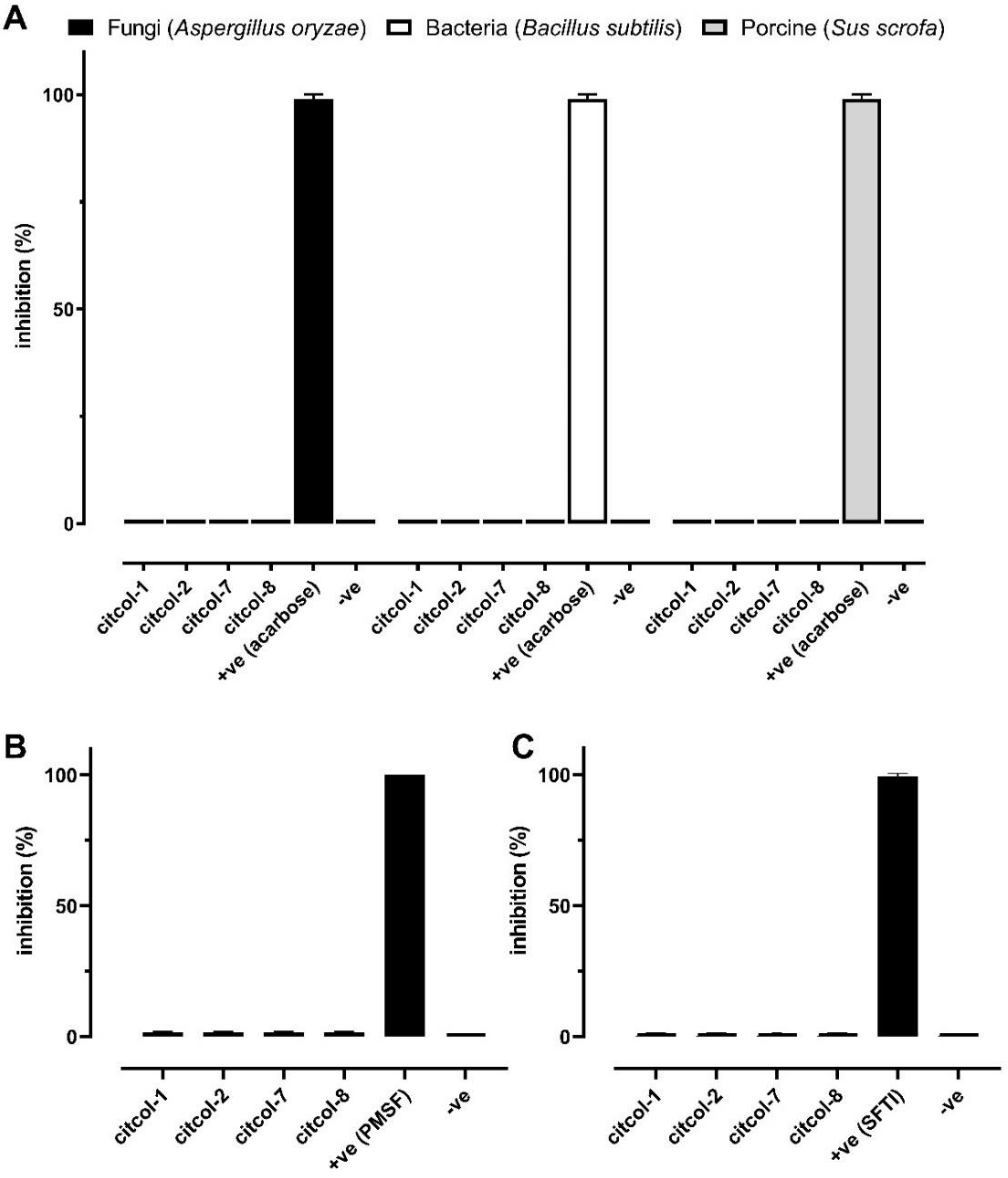

Figure S4. Comparison of active site amino acid in squash family trypsin inhibitory CRPs from Cucurbitaceae (*Momordica spp.*, *Citrullus lanatus*, *Bryonia dioica* and *Cucumis spp.*). Multiple sequence alignment logo generated by using web-based WebLogo application based on online Multiple Sequence Comparison by Log-EXpectation (MUSCLE) tool aligned sequences. The height of each individual residue indicates the relative frequency (in bits) of the residue in that position (as a measure of conservation). The critical amino acids (R and K) in the active site of squash family trypsin inhibitor peptides has been indicated by an orange arrow as P_1 position, which is found to be essential for activity of this type of CRPs [1].

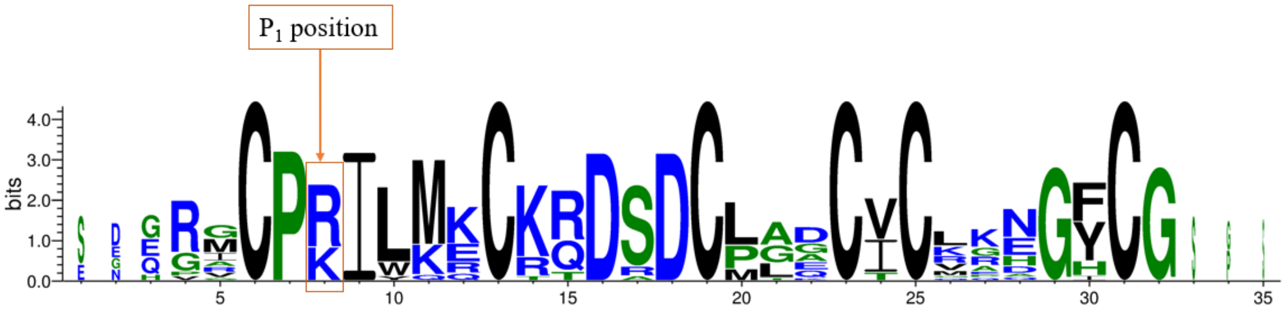

Data 1. Overview and accession codes of genome and transcriptome data (that were used in this study)

Data 2. Cucurbitaceae sequences most similar to citcol peptides that were used for phylogenetic analysis (see Figure 4)

References

1. Krowarsch, D.; Dadlez, M.; Buczek, O.; Krokoszynska, I.; Smalas, A.O.; Otlewski, J. Interscaffolding additivity: binding of P1 variants of bovine pancreatic trypsin inhibitor to four serine proteases. *Journal of Molecular Biology* **1999**, *289*, 175-186.
